# Management of Iatrogenic Aortic Dissections During Percutaneous Right Coronary Artery Interventions

**DOI:** 10.21470/1678-9741-2020-0570

**Published:** 2022

**Authors:** Marco Gennari, Giorgio Mastroiacovo, Gianluca Polvani, Franco Fabbiocchi, Marco Agrifoglio

**Affiliations:** 1 Department of Cardiovascular Surgery, IRCCS Centro Cardiologico Monzino, Milan, Italy; 2 Department of Interventional Cardiology, IRCCS Centro Cardiologico Monzino, Milan, Italy; 3 Department of Biomedical, Surgical and Dental Sciences, University of Milan, Italy

**Keywords:** Coronary Angiography, Iatrogenic Aortic Dissection, Ascending Aorta Replacement, Coronary Artery Bypass.

## Abstract

Iatrogenic acute aortic dissections during percutaneous coronary interventions are an extremely rare but potentially life-threatening complication, occurring in less than 0.02% of transcatheter procedures. We report three patients with different characteristics suffering from iatrogenic aortic dissection during percutaneous coronary intervention successfully treated with an emergency open-heart surgery. A conservative strategy should be pursuit only in small, localized lesions.

**Table:** 

Abbreviations, Acronyms & Symbols	
CABG	= Coronary artery bypass grafting
CPB	= Cardiopulmonary bypass
CT	= Computed tomography
DES	= Drug-eluting stents
ECMO	= Extracorporeal membrane oxygenation
IAAD	= Iatrogenic acute aortic dissection
PCI	= Percutaneous coronary interventions
PTCA	= Percutaneous transluminal coronary angioplasty
RCA	= Right coronary artery
STEMI	= ST-elevation myocardial infarction

## INTRODUCTION

Iatrogenic acute aortic dissection (IAAD) during percutaneous coronary interventions (PCI) is an extremely rare but potentially life-threatening complication, occurring in < 0,02% of the procedures. Patients with a limited aortic wall involvement have been successfully treated by sealing the entry tear with a coronary stent. Dissections extending up to the aorta > 40 mm from the coronary ostia more often require an emergency surgical intervention^[1]^.

We report three different cases of IAAD successfully treated with ascending aorta replacement.

## CASE PRESENTATION

The first case was a 65-year-old Caucasian female with a past medical history of non-ST elevation myocardial infarction who was referred to our center for a new onset of inferior ST-elevation myocardial infarction (STEMI). She was promptly submitted to percutaneous transluminal coronary angioplasty (PTCA) with implantation of four drug-eluting stents (DES) to the right coronary artery (RCA).

**Fig. 1:**
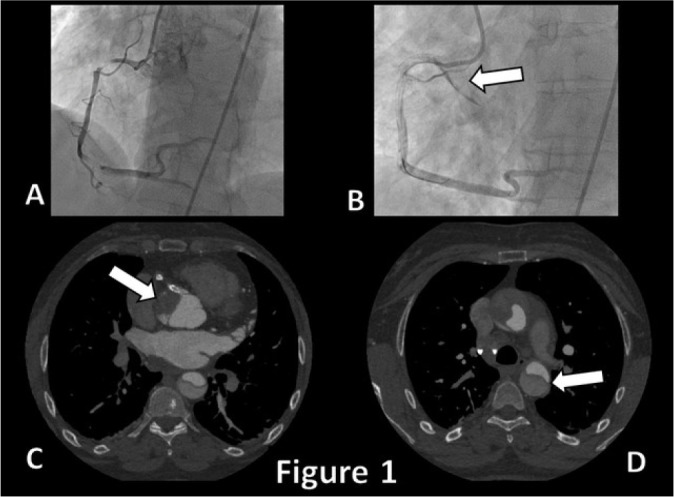
Coronary angiogram showing multiple stenosis on the right coronary artery and the evidence of aortic wall dissection after stenting (A & B, arrow). Axial thoracic computed tomography view showing the type A aortic dissection progressing to the descending aorta (C & D, arrows).

During the procedure, a dissection of the RCA with the subsequent development of an aortic wall hematoma was detected at fluoroscopy; such hematoma quickly extended in a retrograde fashion (Video 1) from the right sinus of Valsalva up to the ascending aorta (Figure 1). An attempt to occlude the intimal entry orifice was made by delivering a right ostial stent, but, unfortunately, the dissection progressed towards the aortic arch, so an emergency surgery was required.

**Video 1:**
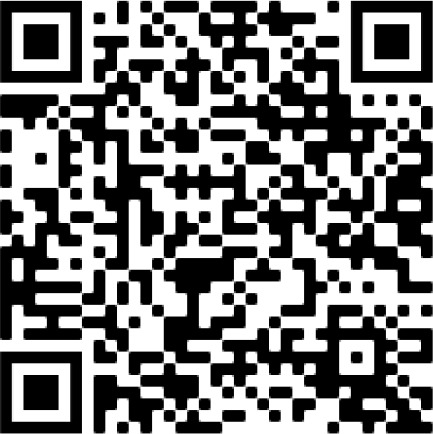
Fluoroscopy of the aortic dissection of case #1.

We performed an ascending aorta replacement with a #28 straight Dacron tube and a single coronary artery bypass grafting (CABG) on the RCA using a saphenous vein graft with a femoro-femoral institution of cardiopulmonary bypass (CPB).

Since the weaning from CPB was unachievable due to hemodynamic instability, an extracorporeal membrane oxygenation (ECMO) implantation was necessary^[2]^.

The patient was finally weaned from ECMO after four days in the intensive care unit. The postoperative course was favorable, and the patient was transferred to a rehabilitation facility.

The second case was a 66-year-old male with a history of STEMI and out-of-hospital cardiac arrest treated with PTCA + DES on the left coronary artery, complicated by the dissection of left anterior descending itself, treated with re-apposition of the stent. During the following years, he was subjected to multiple PTCAs and DES implantations for intra-stent restenosis and de novo ongoing severe lesions on the RCA.

**Fig. 2:**
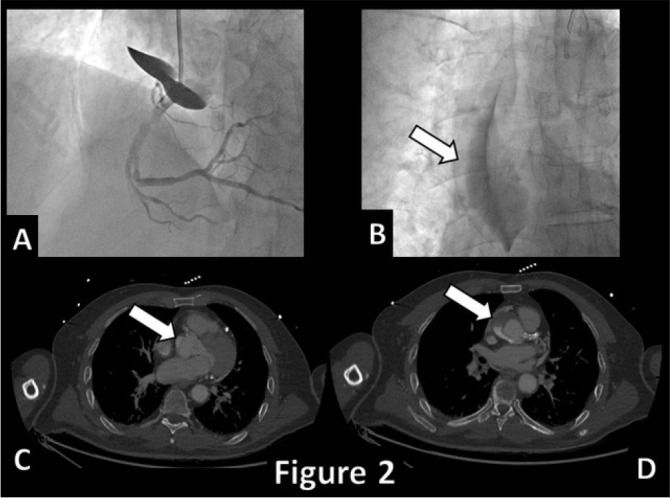
Coronary angiogram with rapid onset and extension of ascending aorta dissection (A & B, arrow) confirmed at an urgent computed tomography scan (C & D, arrows).

During the last procedure, the patient developed a dissection of the proximal part of the RCA (Video 2) that rapidly extended upward to the ascending aorta (Figure 2). An emergency computed tomography (CT) scan showed the progression of the dissection to the innominate and the left common carotid arteries. At the end, he underwent emergency ascending aorta replacement surgery with a #24 Dacron tube prosthesis. The postoperative course was characterized by a minor stroke on the right parietal territory with no clinical remarks at the discharge, on the 7^th^ postoperative day.

**Video 2:**
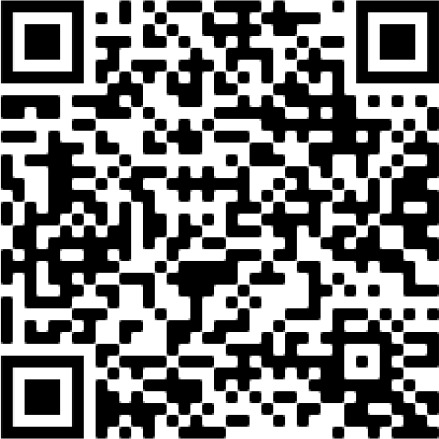
Fluoroscopy of the aortic dissection of case #2.

**Fig. 3:**
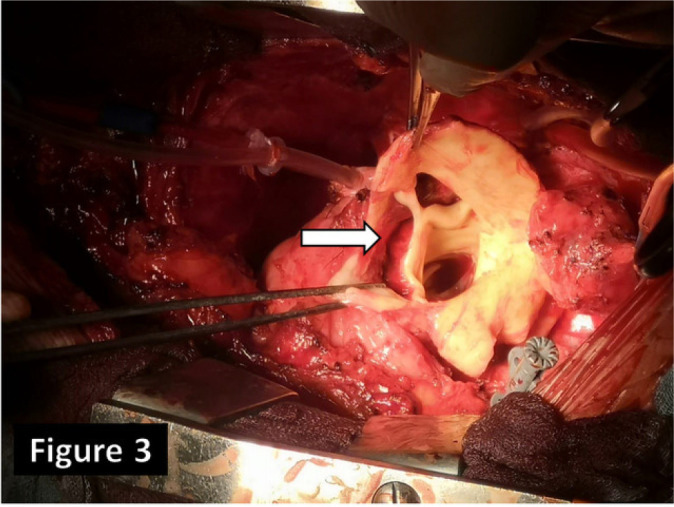
Intraoperative picture. Note the extensive intimal flap within the ascending aorta just above the right coronary ostium towards the non-coronary sinus (arrow).

The last case was a 78-year-old male patient with a history of double emergency CABG for severe instable main stem-related chest pain. During the planned transcatheter complementary revascularization which occurred nearly four months later, an intimal flap of the inner aortic wall near the right coronary ostium was detected after the contrast medium injection (Video 3). The CT scan showed an aortic dissection from the right coronary ostium to the proximal saphenous vein graft anastomosis. The patient underwent an emergency redo surgery (Figure 3) with replacement of ascending aorta with a #28 straight Dacron prosthesis via a femoro-femoral CPB. The patient required the intra-aortic balloon pump and, after weaning, was finally discharged to a rehabilitation center on the 11^th^ postoperative day.

**Video 3:**
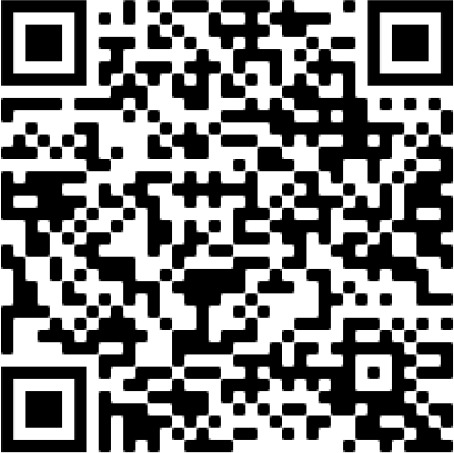
Fluoroscopy of the aortic dissection of case #3.

## DISCUSSION

One of the most fearsome complications that can occur during PCI is an IAAD that could extend in a retrograde fashion to the ascending aorta. A diseased vessel wall with multiple calcifications and atherosclerotic plaques seems to be the most important predisposing factor. In most cases, coronary dissection is easily diagnosed during the coronary angiography which usually reveals a true and a false lumen, separated by a radiolucent intimal flap and a dye staining persistently localized^[3]^.

The process underlying IAAD is not yet completely clear; in fact, there are different mechanisms involved. Firstly, the dissection may be caused by the high-pressure injection of contrast medium on a pre-existing dissection breach. Secondly, shearing forces during systole and diastole could explain the propagation of the dissection in a retrograde manner. Finally, the entry breach could also be created by direct trauma of the angiographic catheters and wires, and increased by forced injection of contrast medium^[4]^.

The type of treatment is different depending on the type and extension of the dissection. An IAAD that remains localized at the level of the Valsalva sinus during the procedure and that extend retrogradely it is preferable to maintain a conservative attitude, as most tend to spontaneously regress with the collaboration of the anterograde aortic blood flow^[5]^.

If the dissection extends < 40 mm into the ascending aorta from the coronary ostia and progresses in an anterograde fashion, then it is preferable to intervene by stenting the affected coronary artery so as to close the breach and prevent the dissection from spreading^[6]^. The third type of strategy consists in an emergency ascending aorta replacement, and it is recommended if the dissection extends > 40 mm from the coronary artery ostium, if the patient is hemodynamically unstable, presents with severe aortic insufficiency, develops hemopericardium, or if the guidewire fails to cross the occluded lesion. Coronary stenting can be useful in these cases as a “bridge to the surgery” and can avoid or reduce the progression of the dissection.

## CONCLUSION

In conclusion, the goal in the treatment of IAAD should be closing the intimal tear as quickly as possible to prevent the progression of dissection and to avoid damage to neurological system and other end organs. A percutaneous attempt is always recommended if suitable, but if it does not achieve a satisfactory result, a prompt ascending aortic replacement is often mandatory.

**Table:** 

Authors’ Roles & Responsibilities	
MG	Substantial contributions to the conception of the work; drafting the work; final approval of the version to be published
GM	Substantial contributions to the acquisition of data for the work; final approval of the version to be published
GP	Final approval of the version to be published
FF	Final approval of the version to be published
MA	Final approval of the version to be published
